# Early Divergence, Broad Distribution, and High Diversity of Animal Chitin Synthases

**DOI:** 10.1093/gbe/evu011

**Published:** 2014-01-16

**Authors:** Anne-C. Zakrzewski, Anne Weigert, Conrad Helm, Marcin Adamski, Maja Adamska, Christoph Bleidorn, Florian Raible, Harald Hausen

**Affiliations:** ^1^Sars International Centre for Marine Molecular Biology, Bergen, Norway; ^2^University of Leipzig, Institute of Biology, Molecular Evolution and Animal Systematics, Germany; ^3^Max F. Perutz Laboratories and Research Platform “Marine Rhythms of Life,” University of Vienna, Austria; ^4^Present address: University College London, Department of Genetics, Evolution and Environment, United Kingdom

**Keywords:** chitin synthase, evolution, Lophotrochozoa, Metazoa, myosin motor domain

## Abstract

Even though chitin is one of the most abundant biopolymers in nature, current knowledge on chitin formation is largely based only on data from fungi and insects. This study reveals unanticipated broad taxonomic distribution and extensive diversification of chitin synthases (CSs) in Metazoa, shedding new light on the relevance of chitin in animals and suggesting unforeseen complexity of chitin synthesis in many groups. We uncovered robust orthologs to insect type CSs in several representatives of deuterostomes, which generally are not thought to possess chitin. This suggests a broader distribution and function of chitin in this branch of the animal kingdom. We characterize a new CS type present not only in basal metazoans such as sponges and cnidarians but also in several bilaterian representatives. The most extensive diversification of CSs took place during emergence of lophotrochozoans, the third large group of protostomes next to arthropods and nematodes, resulting in coexistence of up to ten CS paralogs in molluscs. Independent fusion to different kinds of myosin motor domains in fungi and lophotrochozoans points toward high relevance of CS interaction with the cytoskeleton for fine-tuned chitin secretion. Given the fundamental role that chitin plays in the morphology of many animals, the here presented CS diversification reveals many evolutionary complexities. Our findings strongly suggest a very broad and multifarious occurrence of chitin and question an ancestral role as cuticular component. The molecular mechanisms underlying regulation of animal chitin synthesis are most likely far more complex and diverse than existing data from insects suggest.

## Introduction

Chitin is known to be one of the most abundant biopolymers in nature and occurs in various contexts across a broad range of species. Best known for its strengthening and protective role in body wall cuticles of arthropods and several other invertebrates (Rudall and Kenchington 1973), chitin also forms part of complex hard structures similar to, for example, mollusc radula teeth (Peters and Latka 1986) or annelid chaetae (Picken and Lotmar 1950). Chitin not only serves as a template for several modes of biomineralization in taxa-like sponges (Ehrlich 2010), cnidarians (Bo et al. 2012), and molluscs (Peters 1972) but also lines the insect midgut assisting in digestion (Hegedus et al. 2009) and operating as anti-infectious barrier (Tellam 1996; Lehane 1997). Moreover, it is a component of the cell wall in fungi (Bowman and Free 2006), diatoms (Durkin et al. 2009), and other unicellular eukaryotes (Herth et al. 1977; Mulisch and Hausmann 1989) and has even been reported in Rhizobacteria (Debellé et al. 1992).

Despite the wide and multifarious distribution of chitin in Metazoa, its role in shaping the fungal cell wall (Yarden and Yanofsky 1991; Bowman and Free 2006) and the consequent implications for host infection has dominated interest in this molecule (Bowman and Free 2006). As it has been shown for several organisms as well, the key step of chitin chain elongation is the repeated addition of UDP-GlcNac units to the growing oligosaccharide chain. This process is catalyzed by the enzyme CS, a well-defined glycosyltransferase family 2 member (Merzendorfer 2011). Interestingly, fungi typically do not only possess a single CS but also several paralogs that fall into seven distinct classes (Roncero 2002). Functional studies point toward not only complex transcriptional and posttranscriptional regulation, differential expression, but also proper interaction and cooperation of the different CSs during cell cycle, hyphal growth, or septum formation (Roncero 2002; Lee et al. 2004; Rogg et al. 2012). Furthermore, several fungal CS classes were shown to exhibit an N-terminal myosin motor domain (MMD) able to interact with the actin cytoskeleton (Tsuizaki et al. 2009) and highly relevant for intracellular CS trafficking and site specificity of chitin secretion (Schuster et al. 2012).

In contrast to the situation in fungi, far less is known about metazoan CSs, leaving it an open question whether complex strategies of chitin synthesis exist in animals as well. It has been reported that in both insects and nematodes, only two CSs exist, and these are differentially expressed in the cuticle and the peritrophic matrix, and the egg shell, respectively (Veronico et al. 2001; Zhu et al. 2002; Zhang et al. 2005; Merzendorfer 2006). In Metazoa, the presence of an MMD has been reported in a bivalve CS involved in shell production (Weiss et al. 2006). However, it has remained unclear whether this domain architecture is the result of a recent event or whether it dates back to an earlier evolutionary time point. Finally, although several other animal groups are known to possess chitin-producing representatives, a systematic account of their CS inventories is still lacking.

In this study, we use novel and public sequences to present a comprehensive, broadly sampled analysis on metazoan CS evolution and architecture. We provide a solid background for functional and evolutionary studies of animal chitin formation and related processes such as biomineralization. We present the first CS sequences from several animals occupying phylogenetic positions critical to understand the evolution of these enzymes. Consequently, we were able to reconstruct the early divergence of metazoan CSs and found evidence for clades uniting different types with specific domain organization and extensive diversification in some animal groups. Unexpectedly, we find that different myosin types fused independently to CSs in metazoans and fungi and that linkage between CSs and MMDs is a common phenomenon in certain groups. Together, our findings show a complex evolutionary history of CSs and suggest complementary complexity in the mechanisms of chitin synthesis in metazoans.

## Results and Discussion

**Fig. 1.—:**
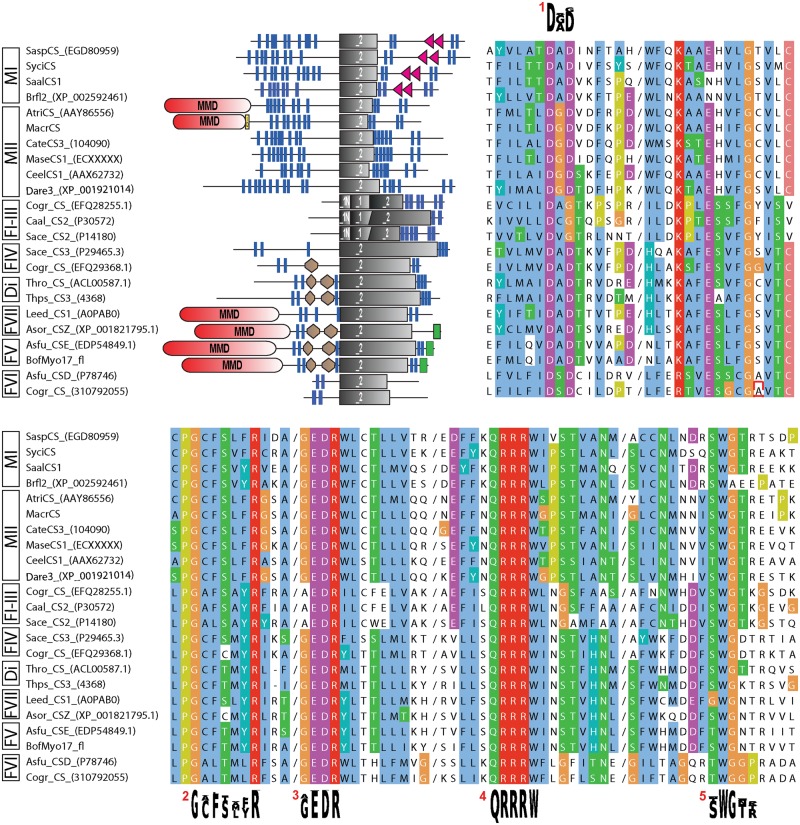
Overview of protein domain architecture and sequence characteristics of a subsample of the analyzed CSs. Clipped alignment (clustalx coloring; cuts indicated by a slash) showing conserved glycosyltransferase family 2 and CS motifs (consensus sequence shown as sequence logos). 1/2, donor saccharide binding; 3, acceptor saccharide binding; 4, product binding; 5, CS-specific motif (possibly involved in chitin translocation). Brown hexagon, Cyt-b5 (Cytochrome b5-like heme/steroid-binding domain); gray rectangle, Pfam CS domains (_1N: Chitin_synth_1N, _1: Chitin_synth_1, _2: Chitin_synth_2); green rectangle, C terminal domain of chromatin-associated protein DEK; MMD (red), myosin motor domain; red triangle, SAM domain; yellow box, IQ domain. Di, diaotome CSs; F1-VII, fungal CSs classes I–VII; MI/C, metazoan type I and choanoflagellate CSs; MII, metazoan type II CSs. For abbreviations of species and protein references, see supplementary table S2, Supplementary Material online.

To obtain a balanced set of CS sequences for gene-tree inference, we mined numerous public sequence resources across Metazoa, fungi, and some protists and novel transcriptomic and genomic data from lophotrochozoan and sponge species. CS identity was confirmed by overall sequence similarity, presence of CS-specific domain architecture, glycosyltransferase family 2, and CS-specific motifs (i.e., donor saccharide-binding, acceptor saccharide-binding, and product-binding motifs) (see fig. 1 and Materials and Methods). Notably, for most species, more than one CS sequence was found (exceptions include, e.g., *Ciona intestinalis* and *Monosiga brevicollis*). Several regular CSs were even recovered in sequence resources of tunicates and vertebrates, from which chitin has been assumed to be absent at all or has been only exceptionally reported (Wagner et al. 1993). Within Metazoa, lophotrochozoans turned out to exhibit the largest pool of CSs with a maximum of ten in the gastropod species *Lottia gigantea*. We further retrieved bona fide CS protein predictions or transcriptomic sequences of several basal-branching metazoans such as calcareous sponges and anthozoans. For these taxa hitherto, no CSs but partly the presence of chitin has been reported (Wilfert and Peters 1969; Ehrlich et al. 2007a, 2007b; Bo et al. 2012). Additional sequences were uncovered from choanoflagellates, the putative sister group of the metazoans.

All obtained sequences share a conserved core region (featuring the Pfam Chitin_synth_2 domain), but in other regions, domain architecture may vary between different kinds of CSs (fig. 1).

**Fig. 2.—:**
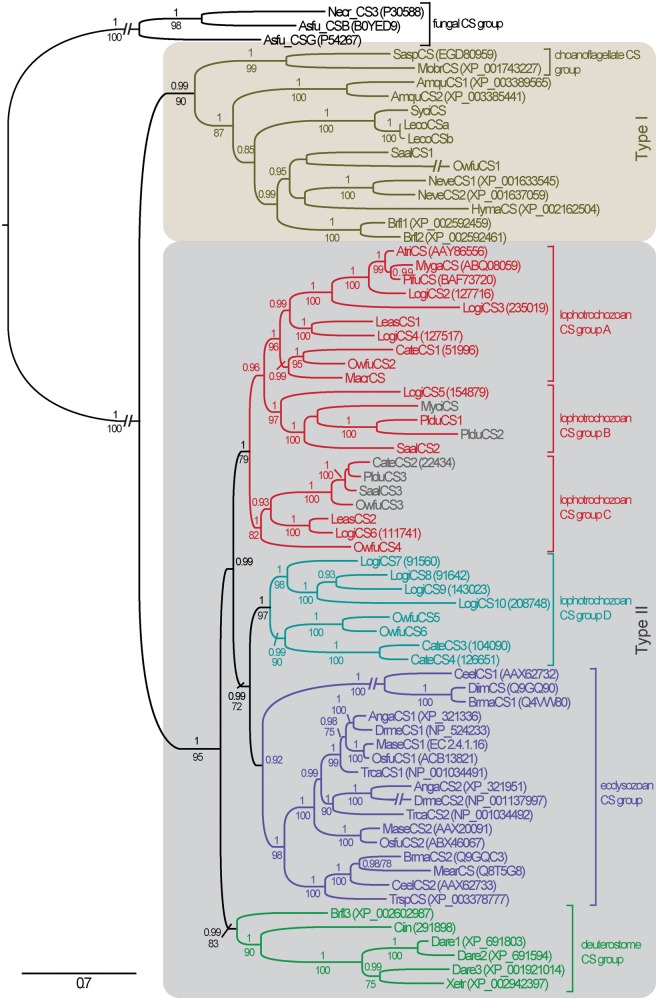
Evolution of metazoan and choanoflagellate CSs with fungal classes I–III CSs as outgroup. Majority rule consensus tree of Bayesian analysis (PhyloBayes, four chains, LG model) based on 926 alignment positions of 71 AA sequences. Only PP values ≥ 0.80 and BS values ≥ 70 of parallel ML analysis (RAxML, LG model, and 1,000 replicates) are shown. Secondary losses of the MMD (occurring within Lophotrochozoa group B and C) are indicated by gray-highlighted CS representatives. The scale bar is in units of amino acid substitutions per site. For abbreviations of species and protein references, see supplementary table S2, Supplementary Material online.

**Fig. 3.—:**
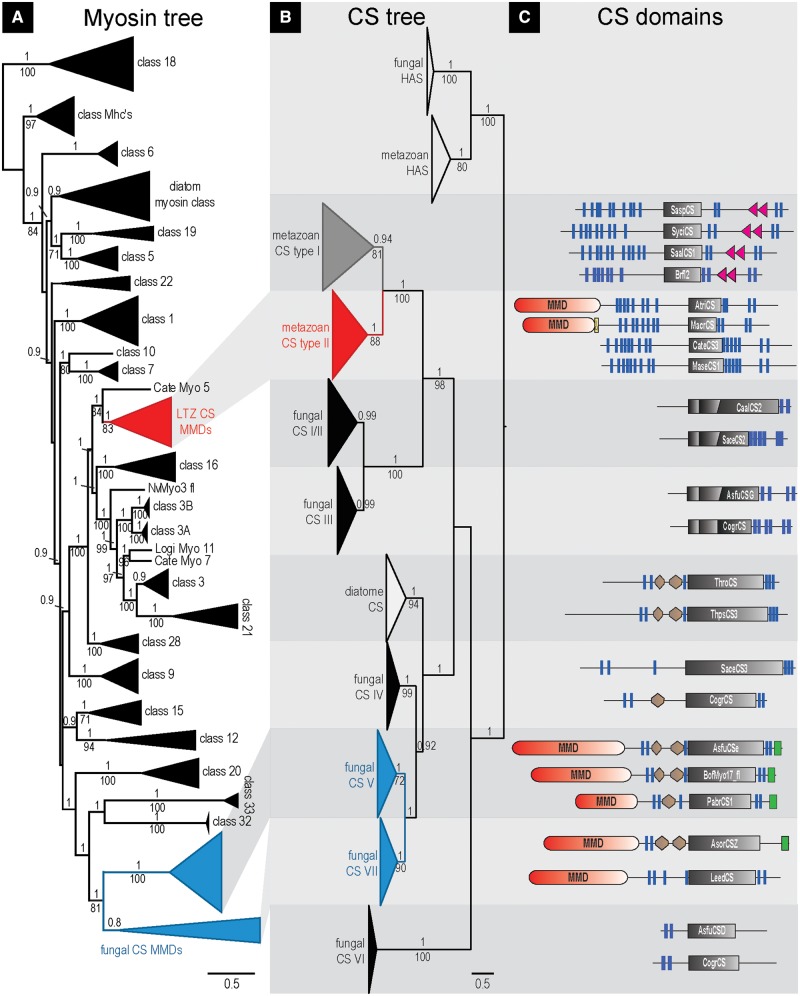
Interrelationships of fungal, diatome, and metazoan CSs and comparison of myosin and CS evolution. (*A*/*B*) Majority rule consensus tree of Bayesian analysis (PhyloBayes, 3/4 chains, LG model) based on 305/568 alignment positions of 127/153 CS/myosin AA sequences. Only PP values ≥ 0.80 and BS values ≥ 70 of parallel ML analysis (RAxML, LG model, 1,000 replicates) are shown. The scale bar is in units of amino acid substitutions per site. (*C*) SMART CS domain predictions of selected CS sequences (sequence name given in each Chitin_synth_2 domain). Transmembrane domains (TMHs; blue bars) are based on the TMHMM 2.0 prediction (only TMH predictions not overlapping other SMART predicted domains are shown). Brown hexagon, Cyt-b5 (Cytochrome b5-like heme/steroid-binding domain); gray rectangle, CS domain; green rectangle, C-terminal DEK_C domain (C terminal domain of chromatin-associated protein DEK); MMD (red), myosin motor domain; red triangle, SAM domain; yellow box, IQ domain.

Metazoan and choanoflagellate CSs, for instance, show conserved patterns of several transmembrane domains on the N- and the C-terminal side of the CS domain. This whole region with exception of the C-terminal sterile alpha motif (SAM) present in some sequences was used to infer the evolution of metazoan CSs, yielding an alignment length of 926 positions (supplementary fig. S3, Supplementary Material online). Maximum likelihood (ML) and Bayesian analyses revealed that metazoan and choanoflagellate CSs can be divided into two major clades (fig. 2). This implies an ancient branching event that is also corroborated by a second, more general analysis of CS evolution including a broad sampling of fungal and diatome sequences (fig. 3*B*).

A closer investigation of the two clades shows that all sponge, cnidarian, and choanoflagellate CSs fall into the type I clade. In addition, we classify two recently published *Branchiostoma* (Guerriero 2012) and some novel lophotrochozoan CSs as type I, confirming the general existence of this CS type across Metazoa. Despite the lack of functional studies on type I CSs, the absence of other CS types in sponges and cnidarians and the existence of chitin in representatives of these groups (Wilfert and Peters 1969; Ehrlich et al. 2007a, 2007b; Bo et al. 2012) suggest that type I CSs can synthesize chitin. In contrast to all other CSs, most metazoan type I CSs exhibit one or two SAM domains in the C-terminal region (fig. 3*C*), suggesting that protein–protein interactions may play an important functional role.

All remaining metazoan CSs fall into group II (figs. 2 and 3*B*). This group includes several predicted but not yet annotated protein sequences from deuterostomes (*Branchiostoma*, *Ciona*, *Danio,* and *Xenopus*). Sequence motifs, domain organization, and phylogeny nevertheless clearly support classification as CSs and suggest that chitin is more widely distributed in Deuterostomia than previously thought. The deuterostome type II CSs differ significantly from the *Branchiostoma* type I CSs reported by Guerriero (2012). Furthermore, they do not correspond to the *Xenopus* DG42 protein and its other vertebrate orthologs, which were reported to synthesize chitin oligomeres with signaling function in early development (Semino et al. 1996), but which later were classified as hyaluronan synthases (Spicer and McDonald 1998; Weigel and DeAngelis 2007). As a result, these uncovered deuterostome sequences turned out to be orthologs of protostome type II CSs, including the well-investigated insect, nematode, and most lophotrochozoan CSs (fig. 2). This strongly appeals for comparative functional investigations, as chitin has been reported in deuterostomes so far only in early studies from fish epidermis (Wagner et al. 1993).

Furthermore, our data support the general existence of two CSs in arthropod and nematode species. In accordance with Zhu et al. (2002), all nematode CS-1 genes share a common origin as do the nematode CS-2 genes. This is seemingly not the case in arthropods. In congruence with Merzendorfer (2006, 2011), all insect CS-1 genes (also referred as class A) form a well-defined clade. CSs classified as class B, however, represent a paraphyletic assemblage, pointing to a more complex evolution of arthropod CSs than hitherto anticipated, including secondary losses in several lineages.

The highest CS diversity within Metazoa exhibits the protostome subgroup of lophotrochozoans. Nearly all sampled representatives were shown to possess several copies of both MMD-containing and nonlinked CSs. The latter forms a well-supported clade (group D in fig. 2 and supplementary fig. S1, Supplementary Material online). MMD-linked CSs, which were in metazoans hitherto only reported from bivalve CSs (Weiss et al. 2006), form three clades of common origin (groups A–C). As suggested by CS tree topology and congruent to the fact that *Lottia* CS2–4 and *Lottia* CS7–8 are tandemly repeated on the genome assembly scaffolds recently published by Simakov et al. (2012), the extraordinarily high number of ten CS copies in *L. gigantea* results from duplication events during early mollusc evolution in Lophotrochozoa group A and maybe even snail evolution in group D.

In this context, alternate exon usage might be a further source for CS diversity and functional variability in lophotrochozoans and other animals as well, as this has already been shown in ecdysozoans (e.g., Arakane et al. 2004).

**Fig. 4.—:**
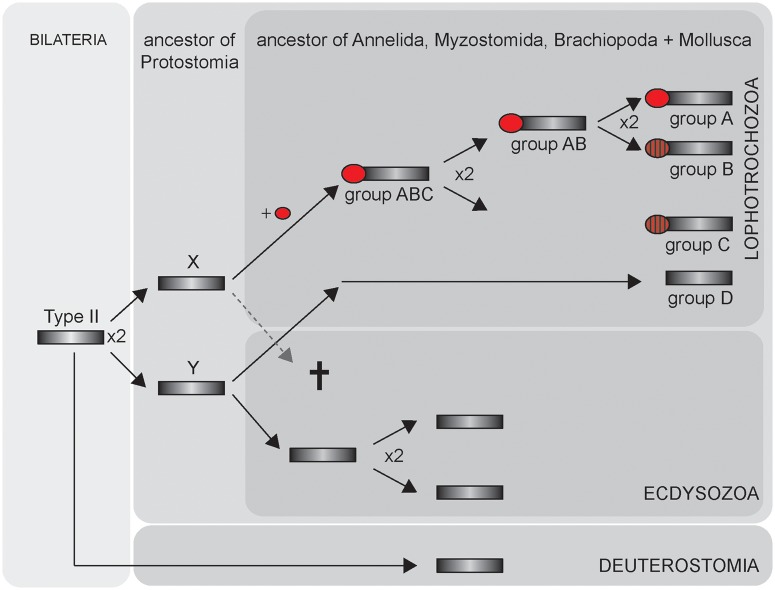
Evolutionary scenario of type II CSs in Bilateria. Dark gray rectangle, CS; red circle, MMD (secondary loss indicated by ruled circles). Gene duplication events are marked with x2.

Nevertheless, diversification of lophotrochozoan CSs started obviously much earlier, and here, our data contribute to the long lasting discussion about the ancestral character set and character evolution of lophotrochozoans. Because all four clades of lophotrochozoan CSs (A-D) contain sequences from different lophotrochozoans, that is, molluscs, brachiopods, myzostomids, and annelids, it is parsimonious to assume that their last common ancestor had already four CS copies: three with and one without MMD (fig. 4). Taking into account that the phylogeny of lophotrochozoans is still not finally resolved (e.g., Helmkampf et al. 2008; Edgecombe et al. 2011), the origin of these genes may even date back to the last common ancestor of Lophotrochozoa. Consequently, because of the extensive CS diversification and the presence of various chitinous structures, lophotrochozoans may be the most interesting taxon to study CS regulatory mechanisms, synergism, and functional divergence in metazoans.

In the context of protein evolution, domain shuffling is widely accepted to be an important driving force and may well explain the composite organization of certain CSs (figs. 1 and 3*C*). To explore the evolutionary origin of the N-terminal MMD present not only in certain lophotrochozoans but also in fungal CSs, we included a broad sampling of fungal and also diatome sequences for a second analysis (fig. 3*B,* supplementary fig. S1, Supplementary Material online). Because of sequence diversity, the alignment had to be restricted to the omnipresent Chitin_synth2 Pfam domain area (supplementary fig. S3, Supplementary Material online). Accordingly, in-group relationships of metazoan type II CSs are less resolved than in the first analysis, although monophyly of metazoan plus choanoflagellate and integrity of metazoan type I and type II CSs are well corroborated (fig. 3*B*). On the other hand, our analysis of the broad data set clearly indicates ancient diversification of the whole protein family and contradicts a monophyletic origin of fungal CSs. In accordance with analyses on general evolution of fungal CSs (Roncero 2002; Ruiz-Herrera et al. 2002; Ruiz-Herrera and Ortiz-Castellanos 2010; Larson et al. 2011), fungal classes I–III CSs form a well-supported clade, and the fungal MMD bearing classes V and VII group together with the MMD lacking fungal class IV CSs (fig. 3*B*, supplementary fig. S1, Supplementary Material online). However, our results differ with respect to a suggested close relationship of fungal chitin classes IV–V and metazoan CSs by some of the aforementioned analyses, which were not rooted by outgroup sequences (Ruiz-Herrera et al. 2002; Ruiz-Herrera and Ortiz-Castellanos 2010) or include only few metazoan sequences (Roncero 2002). Notably, our analysis clearly indicates that diatome CSs are the closest relatives to the fungal classes IV, V, and VII CSs. Furthermore, our data clearly support a sister group relationship of the MMD lacking fungal classes I–III and metazoan and choanoflagellate CSs.

Notably, parallel analysis from a myosin perspective shows that the CS-MMDs of fungi and of lophotrochozoans emerged from different kinds of myosin (fig. 3*A*, supplementary fig. S2, Supplementary Material online), the former grouping with myosin groups XVII and XXXII + XXXIII *sensu* Odronitz and Kollmar (2007) and the latter with myosins III and XVI. For the linkage of MMD and CS, our data thus strongly suggest at least two independent fusion events in fungi and lophotrochozoans, yielding highly similar products, which is a rare phenomenon (Gough 2005; Forslund et al. 2008). We even cannot rule out a third fusion event, as Durkin et al. (2009) report the presence of MMDs in diatom CSs. However, this could not be corroborated by our own protein domain analyses.

The independent fusion of evolutionary distinct CS and myosin sequences may reflect a general relevance of CS interaction with the cytoskeleton. This notion is further substantiated by the integration of N-terminal microtubule interacting and trafficking domains in oomycete CSs (Guerriero et al. 2010). On the other side, the suggested functional relevance has not prevented secondary loss of the MMD in certain lophotrochozoan CSs (figs. 2, 3*C*, and 4).

With its broad taxon sampling, this study is the hitherto most comprehensive analysis of metazoan CS evolution. We provide evidence of ancient diversification of the whole protein family, as well as more recent diversification in several taxa. The data provide compelling justification to study chitin formation in lineages across Metazoa. The underlying mechanisms of this formation process may be most complex in lophotrochozoans as indicated by the high number of CS copies, MMD linkage, and the versatile functions of chitin in this group.

## Materials and Methods

### Public and Own Sequence Resources

Various sequence resources were screened by similarity searches (Blast and HMMER toolkits) for CS and myosin sequences. GenBank, UniProt, and JGI were screened remotely (see supplementary tables S1 and S2, Supplementary Material online, for full list of taxon sampling, abbreviations, accession numbers, and sequence IDs). In case of *Colletotrichum graminicola* and *Thalassiosira pseudonana,* protein prediction data sets were downloaded from the respective genome project webpages, and local search databases were build.

Several transcriptomic data were created by Illumina RNA-seq. In case of the annelids, *Owenia fusiformis* (pooled total RNA from one larval stage and adult head tissue) and *Sabellaria alveolata* (pooled total RNA from five larval stages), and the mollusc, *Leptochiton asellus* (pooled total RNA from four larval stages), library preparation, sequencing (1 lane 100 bp paired end sequencing on HISEQ 2000 per species), and processing of raw data were performed by the Genomic Core Facility of EMBL Heidelberg. Insert size was estimated based on a preliminary assembly by a custom made perl script. Adapter and quality trimming and final De Bruijn graph based read assembly were performed with CLC Genomics Workbench 5.1 (CLC bio, Århus, Denmark).

In case of *Myzostoma cirriferum*, preparation of the mRNA-library, sequencing, and processing of raw data were conducted at the Max Planck Institute for Evolutionary Anthropology (Leipzig, Germany) as described in Hartmann et al. (2012). For library preparation ,RNA of approximately 100 specimens was used. The assembly was generated using the CLC Genomics Workbench 5.1 (CLC bio, Århus, Denmark). Data used for the assembly comprised reads obtained from Hartmann et al. (2012) and reads of an additional run of the identical library on the Illumina Genome Analyzer IIx with 76 cycles paired end.

Transcriptome and genome resources for two calcaronean sponges, *Sycon ciliatum* and *Leucosolenia complicata*, will be described elsewhere (Adamski M, Fortunato S, Leininger S, Rapp HT, and Adamska M, in preparation). For both species, total RNA was isolated from samples containing a variety of developmental stages. In case of *S. ciliatum*, total RNA was also isolated from swimming larvae, laboratory grown juveniles, and fragments undergoing regeneration. Genomic DNA was isolated from nonreproductive single specimens of both species and *S. ciliatum* juveniles grown in laboratory in semisterile conditions. cDNA and genomic libraries were constructed and sequenced using Illumina technology at The Norwegian High-Throughput Sequencing Centre (all samples except juvenile-derived DNA) and DNA Facility Next Generation Sequencing Service at Iowa State University (*S. ciliatum* juvenile-derived DNA). After assembly, *S. ciliatum* genomic scaffolds and cDNA contigs were identified by aligning with juvenile-derived (and therefore devoid of eukaryotic contaminations) genomic sequences.

CS sequences for the annelid *Platynereis dumerilii* were mainly identified from transcriptomic and genomic data generated in ongoing joint sequencing projects to be published elsewhere. Similarly, the CS of the brachiopod *Macandrevia cranium* was identified from larval cDNA sequenced by the Max Planck Institute for Molecular Genetics (Berlin) in a yet unpublished collaborative project.

Fragments of most CSs from the annelids *P. dumerilii* (*PlduCS1*, *PlduCS2*, and *PlduCS3*)*, Capitella teleta* (*CateCS1* [*51996*], *CateCS2* [*22434*], *CateCS3 [104090]*, and *CateCS4 [126651]*), of *M. cirriferum* (*MyciCS*), and the sponge *S. ciliatum* (*SyciCS*) were cloned from cDNA and subsequently sequenced using Sanger technology. All sequences will be submitted to GenBank.

### Sequence Screening

Amino acid sequences of fungal and lophotrochozoan CSs were used as search queries to screen for CS and lophotrochozoan CS-MMD amino acid sequences and HMM-profile Pfam: PF00063 to screen for myosins. CS respectively myosin identity of the obtained sequences were checked by reciprocal Blast and HMMER searches (phmmer), overall domain architecture, and presence of specific domains and motifs (i.e., GESGAG for myosins, donor saccharide-binding, acceptor saccharide-binding, and product-binding motifs for CS motifs) (fig. 1). Furthermore, many myosin sequences were obtained from a recent publication on myosin evolution (Odronitz and Kollmar 2007). Myosin heads of lophotrochozoan CSs were extracted from their full-length sequences and added to the alignment.

As stated earlier, for some CSs, Blast searches revealed the presence of an N-terminal MMD, whereas others lacked this specific region. As CSs are long proteins, N-terminal regions had to be analyzed carefully. Therefore, 5′-regions of contigs respectively scaffolds were examined for 5′-UTR and stop codons. In addition, different polymerase chain reaction (PCR) approaches [including 5′ RACE (Smarter Race Kit, Clonetech) and degenerate-primed fusion PCRs] were carried to elongate cloned sequences (see earlier) and to further substantiate the presence/absence of an N-terminal MMD.

### Protein Domain Predictions

Domain prediction analyses were performed for all CS representatives using SMART (http://smart.embl.de/smart/set_mode.cgi?NORMAL=1, last accessed January 29, 2014) and the TMHMM server v. 2.0 (http://www.cbs.dtu.dk/services/TMHMM/, last accessed January 29, 2014).

### Phylogenetic Analyses

All amino acid sequences were aligned using MAFFT (version 7, http://mafft.cbrc.jp/alignment/server/, last accessed January 29, 2014) and subsequently manually edited in Jalview (Clamp et al. 2004). To visualize domain boundaries in the CS alignments, all respective sequences were screened for Pfam domains in CLC Genomic Workbench 6.0, and the retrieved information was mapped onto the alignment (supplementary fig. S3, Supplementary Material online).

The analysis on fungal, diatome, and metazoan CS interrelationships (fig. 3*B*) is based only on the Pfam Chitinsynth_2 domain region, whereas the analysis on metazoan and choanoflagellate CSs (fig. 2) is additionally based on transmembrane domain regions flanking the Chitin_synth_2 domain. Regions containing myosin motor, SAM, DEK_C, or Cyt-b5 domains were excluded from the alignment. Only alignment positions with high Jalview quality score had been included in the alignment selection. Lower scored amino acid positions were only kept, if they were flanked by high-quality alignment regions. Positions present in less than 10% (corresponding to fig. 3*B*) or 20% (corresponding to fig. 2) of the taxa were removed. Only in the N-terminal region of the alignment, where some sequences were incomplete, slightly higher proportions of gaps were allowed. Attention was also paid to keep integrity of blocks of hydrophobic amino acids (potential TMHs) and conserved hydrophilic amino acid positions (potential functional sites). This led to an alignment length of 568 positions for the myosin analysis, 305 positions for the broadly sampled CS analysis (with fungal and metazoan hyaluronan synthases as outgroup), and 926 positions for the metazoan and choanoflagellate CS analysis (with fungal classes I–III CSs as outgroup). In the latter case of fungal classes I–III CSs, only alignment positions were kept that matched the Pfam Chitin_synth_2 region. All alignment data are included as .fas files in the supplementary data, Supplementary Material online).

Based on the alignments, evolutionary trees were analyzed with both Bayesian inference and ML. ML analyses were conducted with RAxML v7.3.2 (Stamatakis et al. 2008) using the LG + G + F model of evolution and 1,000 fast bootstrap replicates based on CAT approximation. Bayesian interference was conducted with PhyloBayes v3.3 using the LG model of evolution. Model test was performed with a RAxML-based perl script developed by Alexandros Stamatakis and ProtTest 3 (Darriba et al. 2011). The trees shown are the majority-rule consensus of three (corresponding to fig. 3*B*, supplementary fig. S1, Supplementary Material online) and four (corresponding to fig. 2) converged runs of each 6,000/4,000 generations (fig. 3*B*/fig. 2). Chain comparison (bpcomp) was conducted with a burn-in of 1,000, taking one every five trees, up to the end of each chain.

## Supplementary Material

Supplementary data, figures S1–S3, and tables S1 and S2 are available at *Genome Biology and Evolution* online (http://www.gbe.oxfordjournals.org/).

Supplementary Data
