# Supplementary material for: Early Divergence, Broad Distribution, and High Diversity of Animal Chitin Synthases
Source: Genome Biol Evol. 2014 Jan 16;6(2):316–25. doi: 10.1093/gbe/evu011 (PMC3942024; doi:10.1093/gbe/evu011)
Supplement: Supplementary Data [file supp_6_2_316__index.html]

Early divergence, broad distribution and high diversity of animal chitin synthases — Early Divergence, Broad Distribution, and High Diversity of Animal Chitin Synthases — Supplementary Data 

# Early Divergence, Broad Distribution, and High Diversity of Animal Chitin Synthases

## Supplementary Data

files

**Files in this Data Supplement:**

- Supplementary Data - zip file
