# Supplementary figures and images for: Early Divergence, Broad Distribution, and High Diversity of Animal Chitin Synthases
Source: Genome Biol Evol. 2014 Jan 16;6(2):316–25. doi: 10.1093/gbe/evu011 (PMC3942024; doi:10.1093/gbe/evu011)

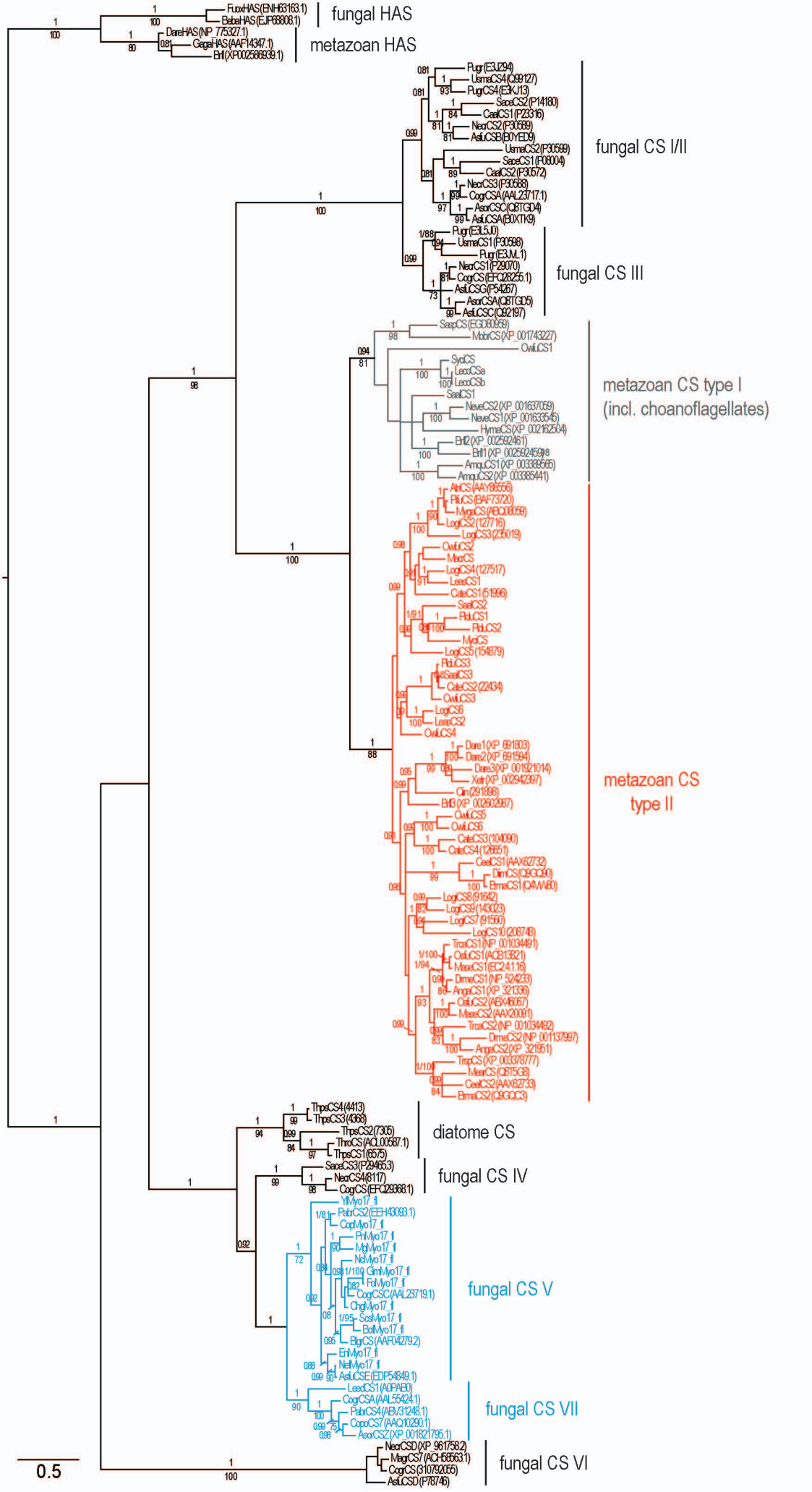

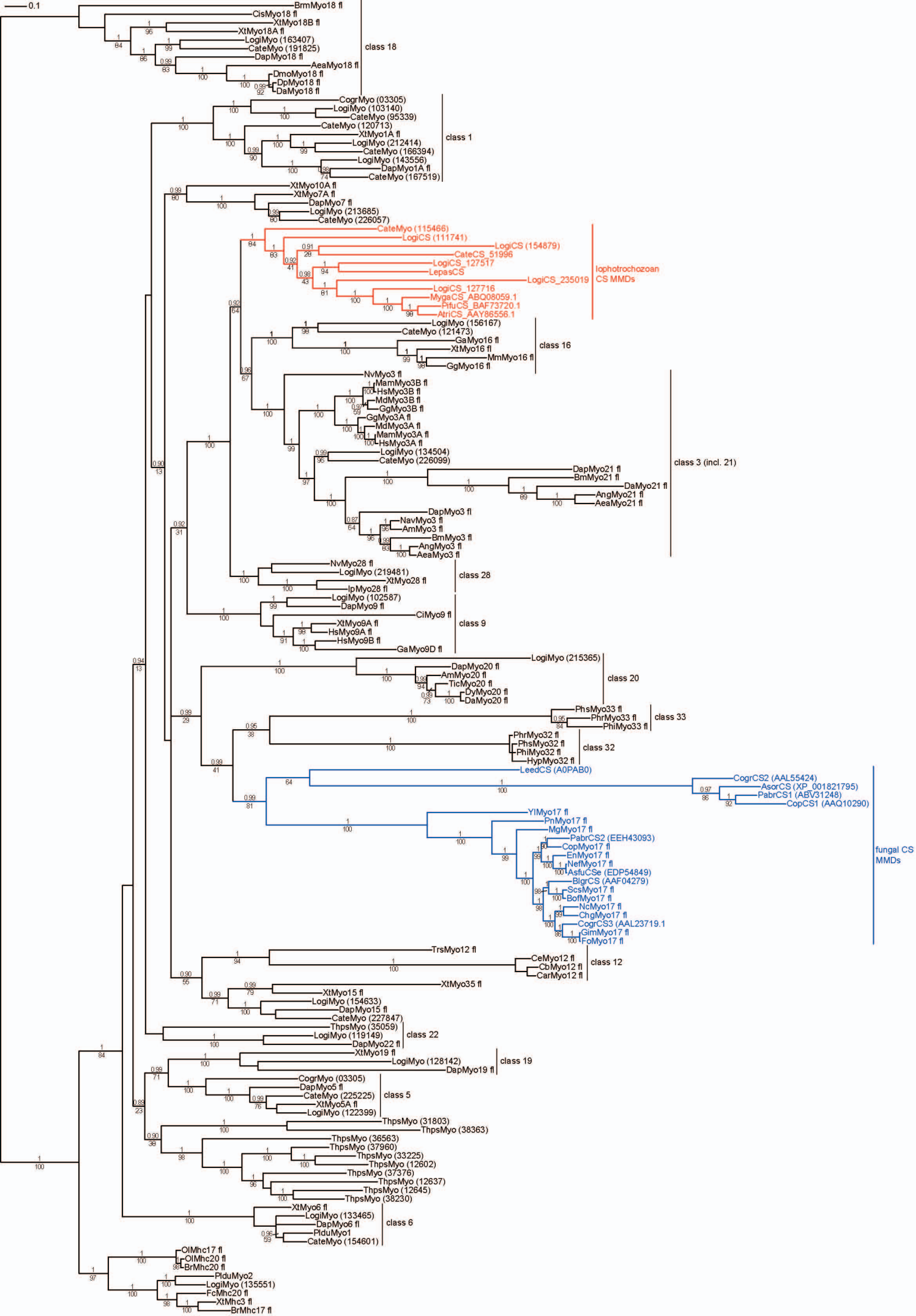

100

### Analysis all CS (Figure 3B)

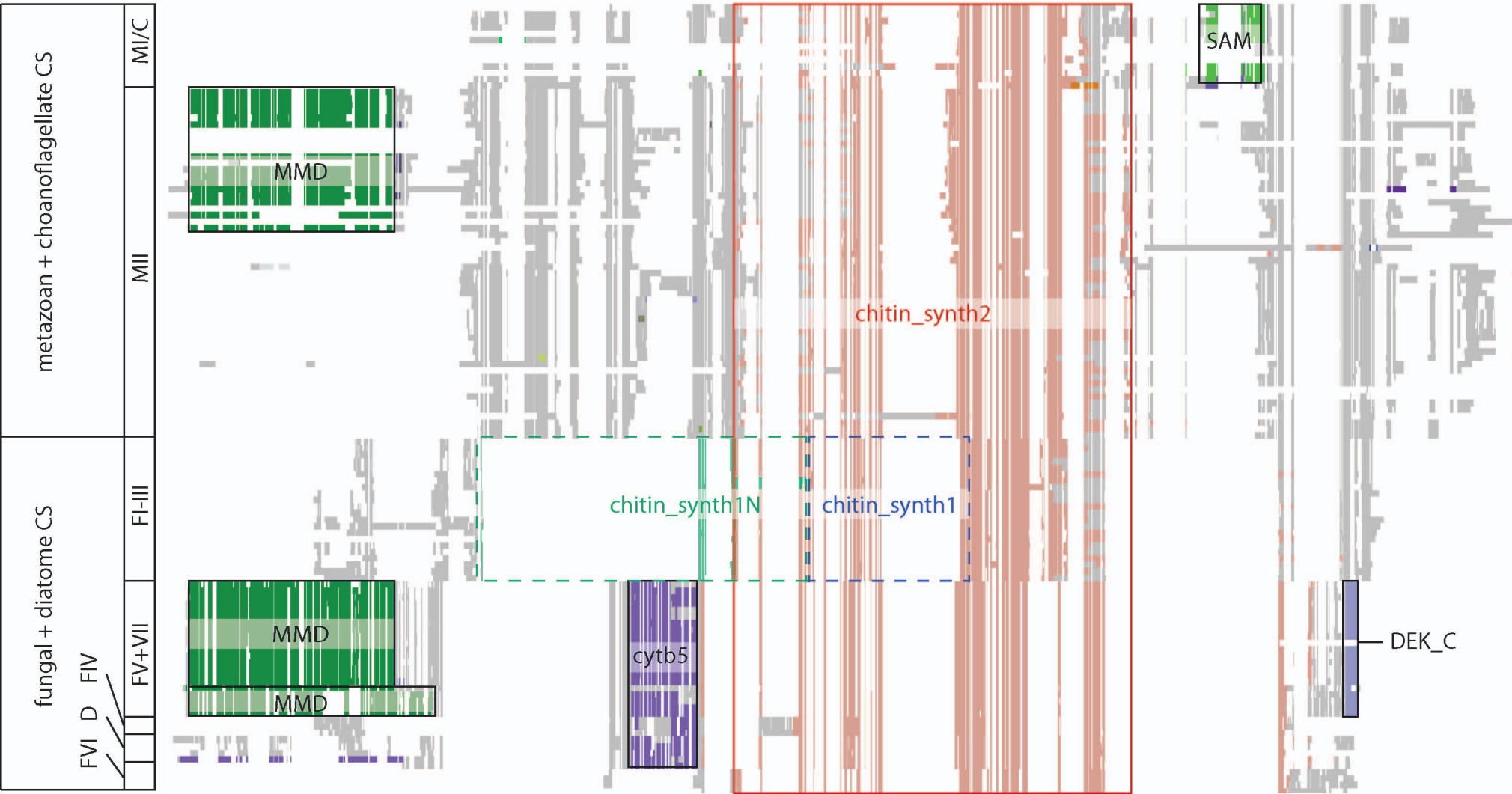

Supplement: Supplementary Data [file supp_evu011_suppl_data.zip › Supplement_Figures.pdf]
